# Supplementary material for: Cervical Cancer Stages, Human Papillomavirus Integration, and Malignant Genetic Mutations: Integrative Analysis of Datasets from Four Different Cohorts
Source: Cancers (Basel). 2023 Nov 26;15(23):5595. doi: 10.3390/cancers15235595 (PMC10705285; doi:10.3390/cancers15235595)
Supplement: Supplementary file 1 [file cancers-15-05595-s001.zip › cancers-2716338-supplementary.pdf]

cancers

MDPI

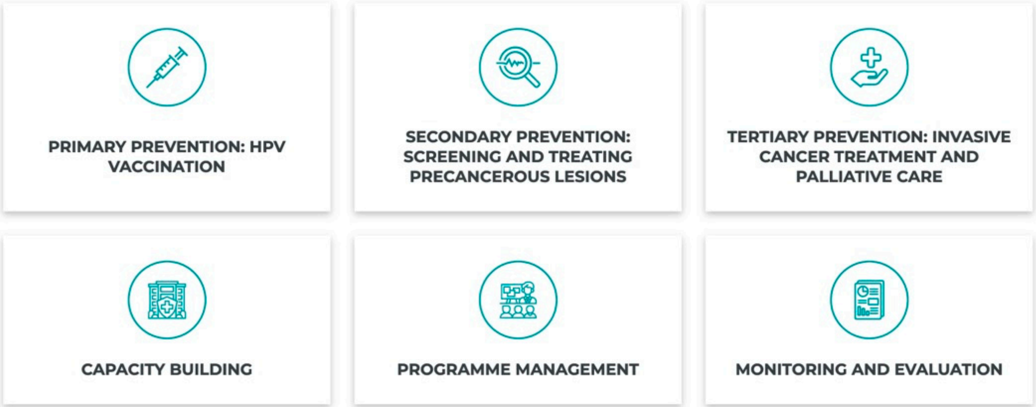


**Figure S1.** World health organization (WHO): Cervical Cancer Elimination Initiative Knowledge Repository (credit: https://cceirepository.who.int/). HPV vaccination is regarded as the primary prevention approach.

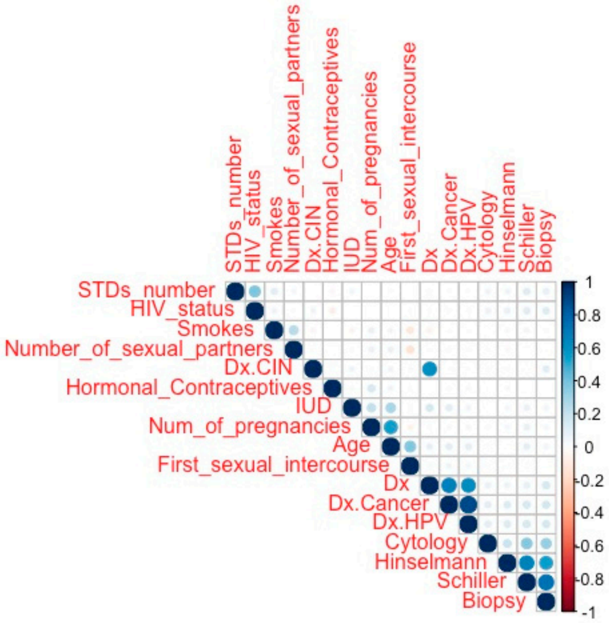


**Figure S2.** Correlation among cervical cancer risk factors. This is based on datasets from the fourth cohort from Caracas, Venezuela.
